# General Control Non-derepressible 1 (AtGCN1) Is Important for Flowering Time, Plant Growth, Seed Development, and the Transcription/Translation of Specific Genes in *Arabidopsis*

**DOI:** 10.3389/fpls.2021.630311

**Published:** 2021-03-31

**Authors:** Xiaona Cui, Kaili Gao, Linjuan Wang, Mengyang Lv, Ziwen Li, Donghua Zheng, Wenwu Wu, Wen Yao, Liying Ding, Xiao Li, Jian-Kang Zhu, Hairong Zhang

**Affiliations:** ^1^College of Life Sciences, Henan Agricultural University, Zhengzhou, China; ^2^Shanghai Center for Plant Stress Biology, Chinese Academy of Sciences, Shanghai, China; ^3^Horticulture and Landscape Architecture, Purdue University, West Lafayette, IN, United States

**Keywords:** protein translation, eIF2α phosphorylation, GCN2, AtGCN1, cold stress

## Abstract

We have previously demonstrated that General Control Non-derepressible 1 (AtGCN1) is essential for translation inhibition under cold stress through interacting with GCN2 to phosphorylate eukaryotic translation initiation factor 2 (eIF2). Here, we report that the flower time of the *atgcn1* mutant is later than that of the wild type (WT), and some siliques of *atgcn1* cannot develop and produce seeds. Total and polysomal RNA of *atgcn1-1* and wild type (WT) after cold treatments were sequenced. The sequencing results show that the mutation of *atgcn1* selectively alters the expression of genes at both transcriptional and translational levels. The classification of AtGCN1 target genes reveals that AtGCN1 regulated gens are involved in flower development, seed dormancy and seed development, response to osmotic stress, amino acid biosynthesis, photosynthesis, cell wall organization, protein transport and localization, lipid biosynthesis, transcription, macroautophagy, proteolysis and cell death. Further analysis of AtGCN1 regulated genes at translational levels shows that the Kozak sequence and uORFs (upstream open reading frame) of transcripts affect translation selection. These results show that AtGCN1 is required for the expression of selective genes in *Arabidopsis.*

## Introduction

Upon stress, the phosphorylation of the alpha subunit of eukaryotic translation initiation factor 2 (eIF2α) is catalyzed by eIF2α kinases to inhibit protein translation in mammals (Erickson and Hannig, 1996). There are four recognized eIF2α kinases in mammals, including a GCN2 (General Control Non-derepressible 2) which is induced under nutrition stress, a double-stranded-RNA-dependent protein kinase R (PKR) which participates in antiviral response, a PKR-like endoplasmic reticulum kinase (PERK) which responds to endoplasmic reticulum stress (ER stress) and a heme-regulated eIF2 inhibitor kinase (HRI) that is activated by heme deprivation. In yeast, however, only one GCN2 kinase has been reported and it is known to get activated by conditions of amino acid deficiency (Zhang et al., 2002; Zaborske et al., 2010), glucose deprivation (Yang et al., 2000), salt stress and UV irradiation (Narasimhan et al., 2004; Wu et al., 2004; Jiang and Wek, 2005).

Sensing amino acid starvation, yeast GCN2 kinase is activated to phosphorylate eIF2α, which results in the inhibition of protein translation (Zhang et al., 2002; Zaborske et al., 2010). Interestingly, eIF2α phosphorylation specifically enhances the translation of transcription factor (TF) *GCN4* in yeast (Dever et al., 1992, 1993; Harding et al., 2000; Lu et al., 2004; Vattem and Wek, 2004), which in turn stimulates the expression of downstream genes to help yeast survive in stress conditions. The downstream genes have been found to involve in amino acid biosynthesis, purine biosynthesis, mitochondrial carrier, peroxisomes, energy metabolism and autophagy (Hinnebusch, 1988; Jia et al., 2000; Natarajan et al., 2001).

In *Arabidopsis*, GCN2 kinase, as the only recognized eIF2α kinase, participates in eIF2α phosphorylation and inhibits protein translation under amino acid deprivation (Lageix et al., 2008). In our previous research, we have found that *Arabidopsis* AtGCN1 interacts with GCN2 to phosphorylate eIF2α upon cold stress, which is essential for the cold-induced inhibition of protein translation and cold acclimation (Wang et al., 2017). However, genes that are regulated by AtGCN1/GCN2 in *Arabidopsis* have yet to be identified. To advance our understanding, we performed total RNA and polysomal RNA sequencing of mutant *atgcn1-1* after cold treatments and found that AtGCN1 mediated eIF2α phosphorylation selectively regulates transcription and translation of genes. Our findings have moved one step closer into understanding the roles of AtGCN1 in *Arabidopsis.*

## Materials and Methods

### Plant Growth Conditions

*Arabidopsis thaliana* ecotype-Columbia, an ethyl methane sulfonate (EMS)-mutagenic mutant *gl1* of Columbia (as the wild type of *atgcn1-1*, WT) and mutants were either grown in forest soil and vermiculite (1:1) or on a half-strength MS medium (M519, Sigma-Aldrich) containing 1% (w/v) of sucrose and 0.8% (w/v) agar at 22°C with a 16 h-light/8 h-dark cycle. To measure root length, fertilized seeds were sown in MS medium with 1% agar and grew for 8 days. For fresh weight measurement, the aboveground parts of four-week-old plants in the soil were cut to be weighed.

### Protein Extraction and Western Blot Analysis

Ten-day-old seedlings were used for protein extraction and proteins were analyzed as described (Wang et al., 2017). The extraction buffer (50 mM Tris-HCl, 150 mM NaCl, 1 mM EDTA, 0.2% Triton X-100, 1 mM DTT, 1 mM PMSF) containing both Complete protease and PhosSTOP phosphatase inhibitor (Roche) was added into the grounded seedlings. The solutions were then centrifuged at 4°C for 5 min twice before extracting proteins from the upper layer. Extracted proteins were incubated at 95°C for 5 min, separated by SDS-PAGE (sodium dodecyl sulphate-polyacrylamide gel electrophoresis) and transferred to PVDF (polyvinylidene fluoride) membranes for blotting. A rabbit monoclonal antibody of phospho-eIF2α (S^51^) (catalog no. 9721, Cell Signaling, 1/1,000 dilution) was probed and then reprobed for Actin (Abmart, 26F7, 1/10000 dilution) to ensure equal loading in each experiment.

### Illumina Sequencing of Total and Polysomal RNA

Ten-day-old seedlings were transferred to cold conditions of 4°C temperature with 16 h-light/8 h-dark cycle for 24 h treatment and used to extract total RNA (TRIzol, Invitrogen) and polysomal RNA. Total and polysomal RNAs were respectively used to construct RNA library and sequenced by Illumina HiSeq2500 in Shanghai Center for Plant Stress Biology (PSC), following standard Illumina protocols (Chen et al., 2016). Two repeats were performed for each treatment.

For polysomal RNA sequencing, whole plants of 10-day-old seedlings after cold treatments were used to extract polysomes as previously reported (Sormani et al., 2011). 200 mg of 10-day-old seedlings was grounded in liquid nitrogen before placing into a 1 ml of lysis buffer (100 mM Tris–HCl, pH 8.4, 50 mM KCl, 25 mM MgCl_2_, 5 mM EGTA, 50 μg/ml cycloheximide, 50 μg/ml chloramphenicol, 0.5% Nonidet P40, Diethyl pyrocarbonate treated) for 10 min at 4°C. After centrifuging at 9,000 *g* for 15 min, the supernatant was loaded onto 12 mL of 0.8-1.5 M sucrose gradients. The gradients were then centrifuged at 175,000 *g* in a Beckman SW41 rotor for 150 min before getting the OD (260 nm) acquired with an ISCO gradient fractionator. Meanwhile, the gradients were fractionated into 21 tubes. According to the OD value at 260 nm, tubes 13-21 containing polysomes were collected together for polysomal RNA extraction using phenol extraction.

For the differentially expressed genes, a threshold of *q*-Value < 0.05, the sum of two signal values > 10 and at least 1.5-fold change were used in the study. Cold-AtGCN1 target genes were filtered out for hierarchical clustering analysis (Cluster 3.0). Functional classification of Cold-AtGCN1 target genes was carried out using DAVID (Database for Annotation, Visualization and Integrated Discovery^1^). The pie charts for Figures 3C,D, 4B,C were drawn in Excel after performing classification.

### RNA Extraction and qPCR

Whole RNA of seedlings treated as the methods in RNA sequencing was extracted according to TRIzol method (Life, Invitrogen). Quantified RNAs were treated with DNase I (Turbo DNA-free kit, Ambion) before getting transcribed into cDNA using oligodT and reverse transcriptase (Goscript Reverse Transcription system;A5001). qPCR analysis was subsequently performed using an IQ5 Multicolor Real-Time PCR Detection system. The following parameters were used to perform qPCR: 95°C for 15 min, 44 cycles of 95°C for 15 s, 55°C for 15s, 72°C for 15 s, followed by a melting curve analysis. Actin was used for normalization and the primers used in qRT-PCR are listed in Supplementary Table 4.

## Results

### *atgcn1* Mutants Flower Later Than the Wild Type

**FIGURE 1:**
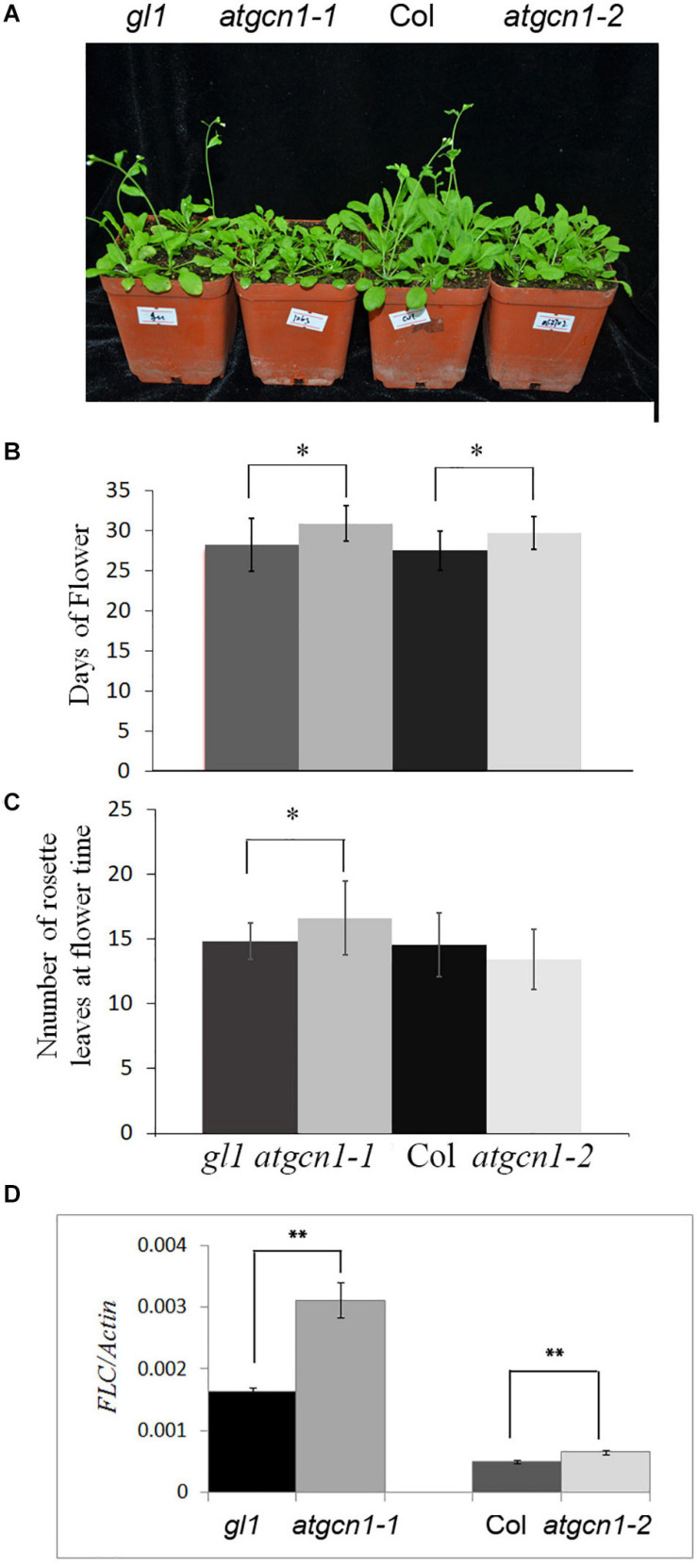
Early flowering of *atgcn1-1* and *atgcn1-2* in soil. **(A)** The flowering phenotype of *atgcn1-1* and *atgcn1-2* in soil. **(B)** The day statistics of flower time. **(C)** The number statistics of rosette leaves at flower time. **(D)** The expression of FLC is higher in *atgcn1-1* and *atgcn1-2* than in WT. Statistical *t*-tests were performed. Single asterisk indicates significant difference with a *p*-Value < 0.05. Double asterisks indicate a significant difference with a *p*-Value < 0.01. Experiments were repeated three times with similar results achieved.

As showed in the previous results, *atgcn1-1* is a point mutant from G to A, which is a truncated protein of AtGCN1 without the C-terminal, while *atgcn1-2*, on the other hand, was achieved through a T-DNA insertion that led to a truncated protein of AtGCN1 without the N-terminal (Wang et al., 2017). Here, we found that both *atgcn1-1* and *atgcn1-2* mutants flowered 2 days later than the WT under long-day conditions (16 h light/8 h dark) (Figures 1A,B). To exclude the germination difference, all the plants on soil were transferred from 5-day-old seedlings in 1/2 strong MS plates. Under short-day conditions (8 h light/16 h dark), mutants did not show to flower later than the WT (not shown). Moreover, we counted the number of rosette leaves at flower time. The result show that the *atgcn1-1* has more rosette leaves than wild type at flower time (Figure 1C). Another mutant of *atgcn1-2* has similar number of rosette leaves as wild type. FLOWERING LOCUS C (FLC) is a gene that negatively controls flowering (Michaels and Amasino, 1999),and we detected the expression of FLC in *atgcn1*. We found that FLC was up-regulated in *atgcn1-1* and *atgcn1-2*, compared to that in WT (Figure 1D). These findings prove that *atgcn1* mutations lead to a late flowering phenotype in Arabidopsis.

### Mutations of *atgcn1* Influences Plant Growth and Seed Development

**FIGURE 2:**
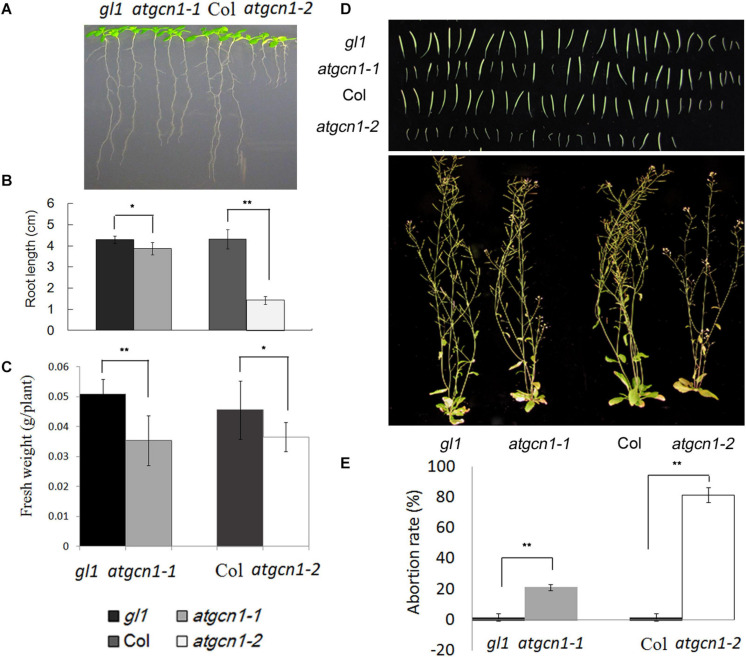
AtGCN1 is required for plant growth and seed development. **(A)** The root length of *atgcn1-1* and *atgcn1-2* is shorter than that of WT. **(B)** The measurement of root length. **(C)** The fresh weight of *atgcn1-1* and *atgcn1-2* is less than that of WT. **(D)** Seed development is impaired by *atgcn1* mutations. **(E)** The statistical analysis of abortive siliques. Statistical *t*-tests were performed. Single asterisk indicates significant difference with a *p*-Value < 0.05. Double asterisks indicate significant difference with a *p*-Value < 0.01. Experiments were repeated three times with similar results attained.

Since mutations of *atgcn1* were reported to impair eIF2 phosphorylation, which has an effect on the translation of mRNAs (Wang et al., 2017), we checked for plant growth alteration in *atgcn1*. The root lengths of *atgcn1* mutants were observed to be significantly shorter than that of WT (Figures 2A,B). Moreover, the weight of rosette leaves in *atgcn1* was evidently lower than that in WT (Figure 2C). These data indicate that the mutation of *atgcn1* inhibits growth in both root and shoot.

In addition to the defect of vegetative growth, we also found that *atgcn1* mutations impaired seed development as partial siliques of mutant plants were observed to be empty (Figures 2D,E). In the *atgcn1-1* mutant line, the rate of aborted siliques is near 20%. In the *atgcn1-2* mutant line, the rate of aborted siliques is around 80%. The results show that *atgcn1* mutations impair the growth at both vegetative and reproductive stages.

As shown in Figure 2, the *atgcn1-2* mutant line has more severe phenotype, with much shorter root length and higher rate of aborted siliques than *atgcn1-1*. The remarkable difference of the vegetative growth and seed production observed between *atgcn1-1* and *atgcn1-2* suggests that the N-terminal of AtGCN1 has a greater impact on plant functions than the C-terminal. The more severe *atgcn1-2* allele has few seeds and it is not enough to be used, so the further experiments are conducted with the less severe *atgcn1-1* allele.

### The Phosphorylation of eIF2α Selectively Regulates Transcription in Cold Stress

**FIGURE 3:**
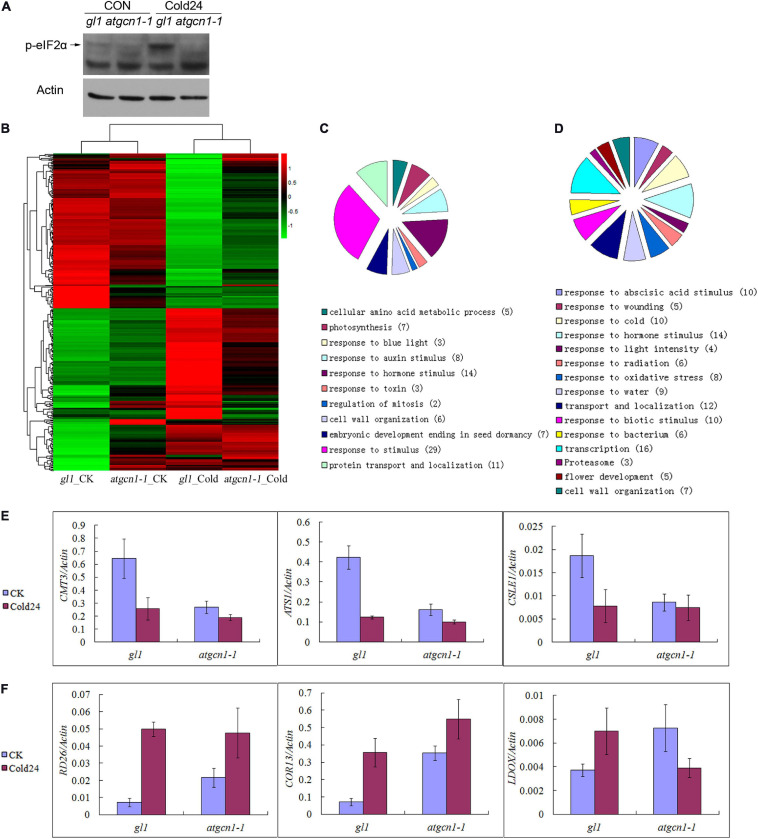
eIF2α phosphorylation selectively regulates gene transcription under cold stress conditions. **(A)** The immunoblotting assays of antibodies against phosphorylated eIF2α under normal temperature and cold stress conditions. Western blot results with Actin used as a control. **(B)** Hierarchical clustering analysis of cold-ATGCN1 genes at transcriptional levels. **(C)** GO terms analysis of cold-ATGCN1 down-regulated genes at transcriptional levels. **(D)** GO terms analysis of cold-ATGCN1 up-regulated genes at transcriptional levels. **(E)** Cold-ATGCN1 down-regulated genes selected for validation by using qRT-PCR. **(F)** Cold-ATGCN1 up-regulated genes selected for validation by using qRT-PCR. CK, grown in normal temperature conditions. Cold or Cold24, treated by cold stress conditions of 4°C temperature for 24 h treatment. qRT-PCR was repeated three times with similar results attained.

AtGCN1 has been reported to interact with GCN2 to phosphorylate eIF2α, which results in translation inhibition under cold stress (Wang et al., 2017). As shown in Figure 3A, eIF2α phosphorylation was induced by cold stress in WT, while the phosphorylation disappeared in *atgcn1-1*. To explore how AtGCN1/GCN2-mediated eIF2α phosphorylation regulates gene expression in *Arabidopsis*, we extracted the total RNA of WT and *atgcn1-1* after cold treatments for a global transcriptome analysis using RNA sequencing.

Based on statistical analysis and after filtering, 184 down-regulated and 179 up-regulated genes were identified and respectively named as cold-AtGCN1 down-regulated or up-regulated genes (Supplementary Table 1). Upon cold stress, these down-regulated or up-regulated genes were either down-regulated or up-regulated at transcriptional levels in WT (the sum of two signal values > 10; a signal ratio fold change ≥ 1.5; *p*-Value < 0.05), whereas the regulation of these genes remains unaltered in *atgcn1-1* (the sum of two signal values > 10; a signal ratio fold change ≤ 1.5).

Cold-AtGCN1 regulated genes at transcriptional levels were then randomly selected for further validation by qRT-PCR. Genes such as CXG methyltransferase choromomethylase 3 (CMT3), chloroplast glycerol-3-phosphate acyltransferase (ATS1) and cellulose synthase like E1 (CSLE1) were discovered to be down-regulated by cold stress in WT, but the expression of these genes was noticeably less or lost in *atgcn1-1* (Figure 3E and Supplementary Table 1). Additionally, TF in response to desiccation (RD26), cystine lyase involved in cysteine and ethylene biosynthesis (CORI3), leucoanthocyanidin dioxygenase involved in proanthocyanin biosynthesis (LDOX) were confirmed to be up-regulated by cold stress in WT, but the regulation was less or lost in *atgcn1-1* (Figure 3F and Supplementary Table 1). The results from qRT-PCR are mostly consistent with those of RNA sequencing data, confirming the efficiency and reliability of our RNA sequencing results.

Our hierarchical clustering analysis of cold-AtGCN1 regulated genes showed that although the repression level was lower in *atgcn1-1* than in WT (Figure 3B and Table 1), most cold-AtGCN1 down-regulated genes was repressed by cold stress in *atgcn1-1.* The result suggests that an alternative mechanism that is independent of eIF2α phosphorylation is involved to affect the transcription of cold-AtGCN1 down-regulated genes. Hierarchical clustering analysis of cold-AtGCN1 up-regulated genes also supports the existence of an alternative mechanism, apart from eIF2α phosphorylation, to induce the expression of cold-AtGCN1 up-regulated genes. As shown, most of the cold-AtGCN1 up-regulated genes were activated by cold stress in *atgcn1-1*, although the induced levels were lower than that in the WT (Figure 3B and Table 2).

**TABLE 1:** Partial classification data of Cold-ATGCN1 down-regulated genes at transcriptional levels.

Local ID	gl1 CK	gl1 cold	Fold change gl1 cold/gl1CK	gcn1-1 CK	gcn1-1cold	Fold change gcn1-1cold/gcn1-1 CK	Full_name
**Cellular amino acid metabolic process 5**							
AT2G35500	**59.44**	**36.24**	−1.64	54.41	46.25	−1.18	SHIKIMATE KINASE-LIKE 2 (SKL2)
AT1G48520	**50.43**	**33.22**	−1.52	48.82	38.20	−1.28	GLU-ADT SUBUNIT B (GATB)
AT1G05010	**263.96**	**155.29**	−1.70	245.05	168.53	−1.45	ETHYLENE-FORMING ENZYME (EFE)
AT5G52100	**38.08**	**21.90**	−1.74	37.64	25.78	−1.46	CHLORORESPIRATION REDUCTION 1 (crr1)
AT3G61440	**307.46**	**177.60**	−1.73	291.31	208.08	−1.40	CYSTEINE SYNTHASE C1 (CYSC1)
**Embryonic development ending in seed dormancy 7**							
AT3G27670	**7.04**	**4.32**	−1.63	−5.70	4.44	−1.28	RESURRECTION1 (RST1)
AT4G24660	**11.93**	**7.44**	−1.60	16.07	13.14	−1.22	HOMEOBOX PROTEIN 22 (HB22)
AT2G41720	**18.60**	**11.66**	−1.59	17.28	13.98	−1.24	EMBRYO DEFECTIVE 2654 (EMB2654)
AT1G69770	**18.27**	**11.71**	−1.56	15.97	12.70	−1.26	CHROMOMETHYLASE 3 (CMT3)
AT5G18700	**6.56**	**4.22**	−1.56	6.43	5.06	−1.27	RUK
AT1G32200	**42.48**	**23.69**	−1.79	36.16	29.22	−1.24	ATS1
AT1G23080	**68.25**	**40.46**	−1.69	61.88	49.27	−1.26	PIN-FORMED 7 (PIN7)

*The signal values of regulated genes are bold, as well as in all the tables.*

**TABLE 2:** Partial classification data of Cold-ATGCN1 up-regulated genes at transcriptional levels.

Local ID	gl1 CK	gl1 cold	Fold change gl1 cold/gl1CK	gcn1-1 CK	gcn1-1cold	Fold change gcn1-1cold/gcn1-1 CK	Full_name
**Response to cold 10**							
AT2G40140	**31.55**	**52.88**	1.68	69.35	47.59	0.69	CZF1
AT5G59820	**11.83**	**29.44**	2.49	36.81	37.05	1.01	RESPONSIVE TO HIGH LIGHT 41 (RHL41)
AT3G05890	**223.74**	**354.57**	1.58	251.30	263.43	1.05	RARE-COLD-INDUCIBLE 2B (RCI2B)
AT5G10140	**2.70**	**4.19**	1.55	7.74	8.41	1.09	FLOWERING LOCUS C (FLC)
AT4G25480	**8.95**	**14.57**	1.63	17.58	14.96	0.85	DEHYDRATION RESPONSE ELEMENT B1A (DREB1A)
AT4G25470	**17.27**	**39.97**	2.31	37.57	30.30	0.81	C-REPEAT/DRE BINDING FACTOR 2 (CBF2)
AT4G02520	**225.38**	**353.50**	1.57	305.74	308.29	1.01	GLUTATHIONE S-TRANSFERASE PHI 2 (GSTF2)
AT2G30250	**12.20**	**22.18**	1.82	19.15	26.00	1.36	WRKY DNA-BINDING PROTEIN 25 (WRKY25)
AT1G76180	**709.28**	**1270.5**	1.79	853.64	1096.7	1.28	EARLY RESPONSE TO DEHYDRATION 14 (ERD14)
AT1G69270	**7.57**	**11.76**	1.55	10.46	11.88	1.14	RECEPTOR-LIKE PROTEIN KINASE 1 (RPK1)
AT2G40140	**31.55**	**52.88**	1.68	69.35	47.59	0.69	CZF1
**Response to wounding 5**							
AT5G59820	**11.83**	**29.44**	2.49	36.81	37.05	1.01	RESPONSIVE TO HIGH LIGHT 41 (RHL41)
AT5G20230	**57.45**	**110.29**	1.92	103.76	91.06	0.88	BLUE-COPPER-BINDING PROTEIN (BCB)
AT4G23600	**45.26**	**77.25**	1.71	85.57	113.48	1.33	CORONATINE INDUCED 1 (CORI3)
AT1G76930	**71.36**	**166.58**	2.33	111.63	152.17	1.36	EXTENSIN 4 (EXT4)
AT4G22880	**2.80**	**7.66**	2.73	4.62	6.83	1.48	LEUCOANTHOCYANIDIN DIOXYGENASE (LDOX)
**Response to light intensity 4**							
AT5G59820	**11.83**	**29.44**	2.49	36.81	37.05	1.01	RESPONSIVE TO HIGH LIGHT 41 (RHL41)
AT5G20230	**57.45**	**110.29**	1.92	103.76	91.06	0.88	BLUE-COPPER-BINDING PROTEIN (BCB)
AT5G59720	**9.18**	**17.51**	1.91	11.67	17.16	1.47	HEAT SHOCK PROTEIN 18.2 (HSP18.2)
AT3G59220	**15.57**	**25.18**	1.62	16.64	21.21	1.27	PIRIN (PRN)

We identified the cold-AtGCN1 regulated genes using DAVID (Database for Annotation, Visualization and Integrated Discovery, See Text footnote 1) (Figures 3C,D and Supplementary Table 1). Five enzymes that involve in amino acid synthesis were down-regulated in WT after cold treatment (Table 1), which was consistent with the fact that fewer amino acids were required for protein translation under cold stress. The five genes were not down-regulated in *atgcn1-1* after cold stress treatment. In addition, seven genes taking part in embryonic development and seed dormancy were down-regulated in WT but not in *atgcn1-1* after cold treatment. Cold-AtGCN1 down-regulated genes were also found to participate in photosynthesis (7), response to blue light (3), response to hormone stimulus (14), response to toxin (3), regulating mitosis (2), cell wall organization (6), and protein transport/localization (11) (Figure 3C and Supplementary Table 1, in the brackets is the number of regulated genes).

As for the cold-AtGCN1 up-regulated genes, we found that a lot of them were involved in response to abiotic stimuli, including abscisic acid, cold, light intensity, oxidative stress and water deprivation (Figure 3D, Table 2, and Supplementary Table 1). These results indicated that AtGCN1 may have potential functional roles in other abiotic stress conditions, in addition to cold stress. Cold-AtGCN1 up-regulated genes were also discovered to involve in protein transport and localization (12), response to biotic stimuli (10), gene transcription (16), proteasome (3), flower development (5) and cell wall organization (7) (Figure 3D and Supplementary Table 1).

### AtGCN1 Selectively Regulates Protein Translation in Cold Stress

As previously mentioned, without eIF2α phosphorylation, polysomes in *atgcn1-1* accumulated slightly higher than that in the WT under cold stress (Wang et al., 2017). To seek out the genes that are regulated by AtGCN1-mediated eIF2α phosphorylation at the translation level, we simultaneously sequenced the polysomal RNA of *atgcn1-1* after cold treatments.

Based on statistical analysis and after filtering, 200 down-regulated and 205 up-regulated genes were identified and respectively named as cold-AtGCN1 down-translated or up-translated genes (Supplementary Table 2). Upon cold stress, the translation of cold-AtGCN1 down-translated or up-translated genes were either down-translated or up-translated in the WT (the sum of two signal values > 10; a fold change of ≥1.5; *p*-Value < 0.05), while the translational regulation of these proteins was not affected in *atgcn1-1* (the sum of two signal values > 10; a fold change of ≤1.5). Additionally, to remove the affection of transcription, we excluded the regulated genes.

**FIGURE 4:**
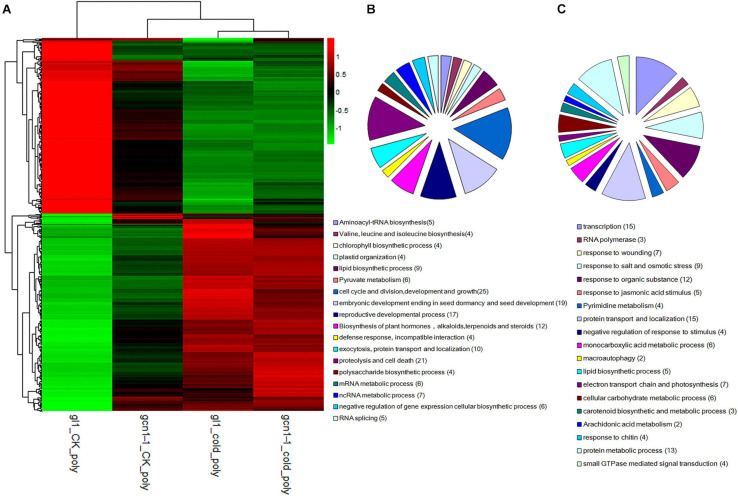
eIF2α phosphorylation selectively regulates protein translation under cold stress conditions. **(A)** Hierarchical clustering analysis of cold-ATGCN1 genes at translational levels. **(B)** GO terms analysis of cold-ATGCN1 genes that were down-regulated at translational levels. **(C)** GO terms analysis of cold-ATGCN1 genes that were up-regulated at translational levels.

Hierarchical clustering analysis of cold-AtGCN1 regulated genes at translational levels demonstrated that most of cold-AtGCN1 inhibited or promoted genes were inhibited or promoted in *atgcn1-1* after cold treatments (Figure 4A and Tables 3, 4), although the regulation level of translation was lower in *atgcn1-1* than in WT. Consistent with the consequence reported for the transcription of regulated genes, the polysomal RNA sequencing data suggest that there are alternative mechanisms involved in regulating protein translation, independent of AtGCN1-mediated eIF2α phosphorylation.

**TABLE 3:** Cold-ATGCN1 down-translated genes are involved in embryonic development ending in seed dormancy and seed development.

Local ID	gl1 CK	gl1 cold	gcn1-1 CK	gcn1-1 cold	gl1 CK _poly	gl1 cold _poly	Fold change gl1 cold_poly/gl1_CK_poly	gcn1-1 CK _poly	gcn1-1 cold _poly	Fold change gcn1-1 cold_poly/gcn1-1 CK_poly	Full_name
AT1G11680	72.35	60.64	65.46	63.40	**8.10**	**4.57**	−1.77	5.73	4.63	−0.82	CYTOCHROME P450 51G1 (CYP51G1)
AT1G13980	12.61	11.07	10.28	9.94	**8.16**	**4.23**	−1.93	5.03	4.17	−0.95	GNOM (GN)
AT1G14610	42.55	56.01	40.31	64.52	**23.82**	**11.85**	−2.01	18.02	12.38	−1.01	TWIN 2 (TWN2)
AT1G36160	29.00	55.48	24.75	62.91	**16.81**	**9.17**	−1.83	11.59	8.61	−0.87	ACETYL-COA CARBOXYLASE 1 (ACC1)
AT1G69040	34.30	27.06	28.79	31.95	**25.35**	**14.05**	−1.80	18.82	16.09	−0.85	ACT DOMAIN REPEAT 4 (ACR4)
AT1G74030	32.46	30.53	29.38	25.09	**28.10**	**14.82**	−1.90	20.31	14.59	−0.92	ENOLASE 1 (ENO1)
AT1G80490	18.40	19.43	15.69	18.47	**10.83**	**5.04**	−2.15	7.04	5.41	−1.10	TOPLESS-RELATED 1 (TPR1)
AT3G48110	29.99	24.44	29.26	32.77	**12.10**	**5.46**	−2.22	11.16	8.13	−1.15	EMBRYO-DEFECTIVE-DEVELOPMENT 1 (EDD1)
AT3G53700	12.95	16.86	11.82	15.47	**9.80**	**5.30**	−1.85	7.72	5.37	−0.89	MATERNAL EFFECT EMBRYO ARREST 40 (MEE40)
AT3G62680	52.87	78.90	58.28	55.45	**11.04**	**5.90**	−1.87	10.61	8.04	−0.90	PROLINE-RICH PROTEIN 3 (PRP3)
AT4G04890	42.58	37.38	36.40	37.39	**18.75**	**10.51**	−1.78	12.70	11.09	−0.83	PROTODERMAL FACTOR 2 (PDF2)
AT4G38600	26.87	40.51	23.01	38.74	**15.45**	**8.54**	−1.81	9.96	7.42	−0.86	KAKTUS (KAK)
AT5G23940	18.01	13.11	14.03	11.92	**7.22**	**3.30**	−2.18	5.98	4.00	−1.13	PERMEABLE LEAVES3 (PEL3)
AT5G26742	219.89	302.05	200.63	340.79	**94.75**	**52.11**	−1.82	75.36	55.93	−0.86	EMBRYO DEFECTIVE 1138 (emb1138)
AT5G37510	74.41	90.16	67.09	84.53	**49.08**	**30.22**	−1.62	34.67	30.52	−0.70	EMBRYO DEFECTIVE 1467 (EMB1467)
AT5G52920	116.19	99.87	91.21	80.00	**58.21**	**34.67**	−1.68	39.41	34.00	−0.75	PLASTIDIC PYRUVATE KINASE BETA SUBUNIT 1 (PKP-BETA1)
AT5G65930	13.75	17.48	12.90	16.21	**8.92**	**4.12**	−2.17	6.21	4.19	−1.12	ZWICHEL (ZWI)

**TABLE 4:** Partial classification data of Cold-ATGCN1 up-translated genes.

Local ID	gl1 CK	gl1 cold	gcn1-1 CK	gcn1-1 cold	gl1 CK _poly	gl1 cold _poly	Fold change gl1 cold_poly/gl1_CK_poly	gcn1-1 CK _poly	gcn1-1 cold _poly	Fold change gcn1-1 cold_poly/gcn1-1 CK_poly	Full_name
**Response to wounding 7**											
AT3G61190	10.72	12.27	22.92	11.24	**2.54**	**8.35**	3.29	11.76	9.77	0.83	BON ASSOCIATION PROTEIN 1 (BAP1)
AT1G72450	25.11	31.94	27.52	45.01	**16.30**	**27.81**	1.71	21.67	30.21	1.39	JASMONATE-ZIM-DOMAIN PROTEIN 6 (JAZ6)
AT5G43580	24.21	19.90	34.47	22.12	**22.69**	**41.33**	1.82	64.76	58.82	0.91	UNUSUAL SERINE PROTEASE INHIBITOR (UPI)
AT1G01720	21.43	29.21	25.91	30.86	**11.12**	**21.17**	1.90	13.81	16.99	1.23	(ATAF1)
AT4G12030	47.76	62.34	60.73	67.97	**18.72**	**30.87**	1.65	23.03	28.45	1.24	BILE ACID TRANSPORTER 5 (BAT5)
AT1G27730	45.75	31.80	114.02	30.55	**16.43**	**31.61**	1.92	85.88	39.00	0.45	SALT TOLERANCE ZINC FINGER (STZ)
AT3G51660	38.80	52.93	46.36	66.32	**37.34**	**102.22**	2.74	83.63	120.45	1.44	
**Response to salt and osmotic stress 9**											
AT3G23600	182.14	149.80	207.23	154.85	**142.88**	**224.40**	1.57	218.87	242.04	1.11	
AT2G38710	39.46	36.35	39.67	29.27	**18.05**	**31.89**	1.77	21.66	30.11	1.39	
AT3G28910	20.41	18.46	19.73	17.58	**7.29**	**13.85**	1.90	12.26	15.35	1.25	MYB DOMAIN PROTEIN 30 (MYB30)
AT5G09650	237.02	195.12	249.44	210.21	**151.88**	**256.86**	1.69	211.18	267.74	1.27	PYROPHOSPHORYLASE 6 (PPa6)
AT2G45790	85.63	69.81	88.05	63.16	**55.32**	**94.85**	1.71	67.44	94.39	1.40	PHOSPHOMANNOMUTASE (PMM)
AT5G67300	55.84	66.86	81.58	60.21	**24.45**	**43.21**	1.77	47.79	42.08	0.88	MYB DOMAIN PROTEIN R1 (MYBR1)
AT1G27730	45.75	31.80	114.02	30.55	**16.43**	**31.61**	1.92	85.88	39.00	0.45	SALT TOLERANCE ZINC FINGER (STZ)
AT1G53580	55.87	63.31	62.81	60.68	**31.23**	**58.80**	1.88	39.10	53.18	1.36	GLYOXALASE II 3 (GLY3)
AT2G41010	12.28	13.75	19.90	16.46	**6.63**	**15.56**	2.35	12.42	17.06	1.37	CALMODULIN (CAM)-BINDING PROTEIN OF 25 KDA (CAMBP25)
**Response to jasmonic acid stimulus 5**											
AT3G28910	20.41	18.46	19.73	17.58	**7.29**	**13.85**	1.90	12.26	15.35	1.25	MYB DOMAIN PROTEIN 30 (MYB30)
AT1G72450	25.11	31.94	27.52	45.01	**16.30**	**27.81**	1.71	21.67	30.21	1.39	JASMONATE-ZIM-DOMAIN PROTEIN 6 (JAZ6)
AT5G67300	55.84	66.86	81.58	60.21	**24.45**	**43.21**	1.77	47.79	42.08	0.88	MYB DOMAIN PROTEIN R1 (MYBR1)
AT3G15210	41.86	33.10	69.23	32.45	**14.83**	**25.94**	1.75	27.23	33.02	1.21	ETHYLENE RESPONSIVE ELEMENT BINDING FACTOR 4 (ERF4)
AT4G12030	47.76	62.34	60.73	67.97	**18.72**	**30.87**	1.65	23.03	28.45	1.24	BILE ACID TRANSPORTER 5 (BAT5)

We classified the cold-AtGCN1 down-translated or up-translated genes using DAVID (Database for Annotation, Visualization and Integrated Discovery, see text footnote 1) (Figures 4B,C and Supplementary Table 2). 19 genes involved in embryonic development ending in seed dormancy and seed development were down-translated by cold stress in WT, due to cold-induced eIF2α phosphorylation (Table 3). On the other hand, they are not regulated in *atgcn1-1*, in which eIF2α phosphorylation is lost. Cold-AtGCN1 down-translated genes were also identified with roles in aminoacyl-tRNA biosynthesis (5), chlorophyll biosynthetic process (4), plastid organization (4), lipid biosynthetic process (9), pyruvate metabolism (6), cell cycle and division, development and growth (25), reproductive developmental process (17), exocytosis, protein transport and localization (10), proteolysis and cell death (21), polysaccharide biosynthetic process (4), mRNA metabolic process (6), ncRNA metabolic process (7), negative regulation of gene expression of cellular biosynthetic process (6), RNA splicing (5), and incompatible interaction with defense response(4) (Figure 4B and Supplementary Table 2).

Lots of genes that were induced in the WT were noted to remain unchanged at translational levels in *atgcn1-1* under cold stress, which are referred as cold-AtGCN1 up-translated genes. Classification results show that cold-AtGCN1 up-translated genes are involved in response to wounding, salt and osmotic stress, jasmonic acid (JA) stimulus (Table 4). Moreover, these genes take part in DNA-directed RNA polymerase (3), response to organic substance (12), pyrimidine metabolism (4), protein transport and localization (15), negative regulation of genes in response to stimulus (4), monocarboxylic acid metabolic process (6), macroautophagy (2), lipid biosynthetic process (5), electron transport chain and photosynthesis (7), cellular carbohydrate metabolic process (6), carotenoid biosynthetic and metabolic process (3), arachidonic acid metabolism (2), response to chitin (4), protein metabolic process (13), small GTPase mediated signal transduction (4), and gene transcription (15) (Figure 4C and Supplementary Table 2).

### Kozak Sequence and uORFs Affect Translation Selection Under Cold Stress Conditions

The initiation codon context is known to be important for translation selection of a mRNA (Kozak, 1987; Hinnebusch, 2011), and the optimized sequence is (A/G)nn**ATG**(G) (Kozak sequence), with the initiation codon (bold) and purine residues at −3 and +4. For a “strong” initiation codon, the nucleotides both at positions +4 (i.e., G in the consensus) and −3 (i.e., either A or G in the consensus) match the consensus; An “adequate” initiation codon match only 1 of 2 sites; a “weak” initiation codon match neither.

According to Araport11 on TAIR (https://www.arabidopsis.org), we analyzed the initiation codon of total genes, cold-AtGCN1 down-translated and up-translated genes (Table 5 and Supplementary Table 2). The rate of transcripts with strong, adequate and weak initiation codon of total genes is respectively 34.0%, 44.5%, and 10.6% in *Arabidopsis*. Among cold-AtGCN1 down-translated genes, 48.4% of the transcripts show a strong start codon and 41.8% of ones have an adequate initiation codon. Among cold-AtGCN1 up-translated genes, 41.3% of the transcripts show a strong start codon and 52.1% of ones have an adequate initiation codon. Thus, the percentage of cold-AtGCN1 down- and up-translated genes with a strong or adequate initiation codon is respectively 90.2 and 93.4%, which are both higher than that in total genes (78.5%). The results show that cold-AtGCN1 mediated eIF2α phosphorylation may regulate the translation preference to transcripts with strong or adequate initiation codon and transcripts with weak initiation codon or without 5′-UTR (untranslated regions, UTR) have low preference to be regulated by cold-AtGCN1 mediated eIF2α phosphorylation.

**TABLE 5:** The frequency of initiation codon and uORFs for cold-AtGCN1 down-translated or up-translated genes.

	Transcripts with strong initiation codon (%)	Transcripts with adequate initiation codon (%)	Transcripts with weak initiation codon (%)	Transcripts without 5′-UTR	uORF frequency in mRNA (%)
Down	48.4	41.8	8.9	0.9	63.4
Up	41.3	52.1	6.6	1.2	58.2
Total genes	34.0	44.5	10.6	10.8	51.2

Yeast GCN4, whose translation is specifically increased in response to eIF2α phosphorylation, has four or two upstream open reading frames (uORFs) prior to the initiation codon (Mueller and Hinnebusch, 1986; Vattem and Wek, 2004). According to Araport11 on TAIR (https://www.arabidopsis.org), we analyzed the uORF of transcripts and the rate of transcripts with uORF was 51.2% (Supplementary Table 2). We hypothesized that eIF2α phosphorylation should promote the translation of transcripts with uORF in plants. However, as shown in Table 1, both 63.4% of down-translated transcripts and 58.2% of up-translated genes with uORFs are higher than 51.2% of total genes with uORFs. The results show that cold-AtGCN1 mediated eIF2α phosphorylation may selectively regulate the translation preference to transcripts with uORFs, which is different from the hypothesis we supposed.

### AtGCN1 Selectively Regulates Gene Transcription and Protein Translation in Normal Temperature Conditions

Hierarchical clustering analysis of cold-AtGCN1 target genes showed that most of them were regulated in *atgcn1-1* in normal temperature conditions (Figures 3B, 4A), suggesting that the low level of eIF2α phosphorylation mediated by AtGCN1 regulates the transcription or translation of specific genes. According to statistics and filtering (the sum of two signal values > 10; fold change ≥ 1.5; *p*-Value < 0.05.), we found 509 genes were up-translated and 249 genes were up-transcribed in *atgcn1-1* in normal temperature conditions, compared to that in WT (Supplementary Table 3). On the other hand, 354 genes were down-translated and 47 genes were down-transcribed in *atgcn1-1*, compared to that in WT in normal temperature conditions. To exclude the effect of transcription, regulated genes at transcriptional levels were eliminated.

We respectively classified the up-translated and up-transcribed genes in *atgcn1-1* using DAVID online. The up-translated genes in *atgcn1-1* include genes responsive to not only abiotic stress including oxidation (16), cold (13), and salt (16), but also hormones including abscisic acid (ABA) (17), ethylene (9), salicylic acid (SA) (11), JA (9), auxin (11), brassinosteroid (BR) (4) and gibberellin (7) (Table 6 and Supplementary Table 3). The up-transcribed genes in *atgcn1-1* are also found involved in abiotic stress and hormone signals including water deprivation (29), cold (19), wounding (24), salt (18), ABA (25), SA (12), JA (12) (Table 7 and Supplementary Table 3). The results show that AtGCN1 mediated eIF2α phosphorylation in normal temperature conditions inhibits the expression of genes involved in stress stimulus and hormone signals, at both transcriptional and translational levels. In addition, up-transcribed genes in *atgcn1-1* are mostly up-regulated at translational levels, and we can’t infer whether the increased translation of these transcripts in *atgcn1-1* contributes to the transcriptional increase or *vice versa*.

**TABLE 6:** Partial classification data of up-translated genes in *atgcn1-1*, compared with that in WT under normal temperature conditions.

Local ID	gl1 CK	gl1 cold	gcn1-1 CK	gcn1-1 cold	gl1 CK _poly	gl1 cold _poly	gcn1-1 CK _poly	gcn1-1 cold _poly	Full_name
**Response to oxidative stress 16**									
AT3G46090	1.52	2.38	6.72	1.47	**2.89**	2.97	**9.19**	2.68	ZAT7
AT4G25100	591.79	392.85	694.79	484.58	**188.67**	437.60	**288.05**	522.18	FE SUPEROXIDE DISMUTASE 1 (FSD1)
AT1G31170	66.68	24.64	77.30	29.25	**36.56**	38.88	**58.42**	41.65	SULFIREDOXIN (SRX)
AT4G03520	558.51	421.36	561.86	433.37	**518.65**	879.52	**798.36**	935.25	ATHM2
AT5G59080	46.37	4.96	54.63	5.59	**26.17**	6.98	**39.43**	12.89	
AT2G25080	415.65	291.65	429.81	299.68	**151.51**	297.67	**232.45**	286.21	GLUTATHIONE PEROXIDASE 1 (GPX1)
AT2G19310	81.45	23.10	81.81	18.66	**42.75**	33.84	**72.68**	32.45	
AT5G07460	62.22	27.95	69.11	35.42	**49.31**	52.01	**78.79**	57.54	PEPTIDEMETHIONINE SULFOXIDE REDUCTASE 2 (PMSR2)
AT2G21640	8.95	8.46	12.98	20.80	**20.39**	21.42	**35.27**	45.78	
AT1G73120	14.78	7.93	10.96	9.86	**12.57**	14.10	**20.97**	15.44	
AT5G56550	36.05	2.85	35.47	2.45	**21.49**	3.37	**35.03**	4.16	OXIDATIVE STRESS 3 (OXS3)
AT3G14430	34.51	22.53	29.38	20.47	**15.50**	45.94	**27.18**	44.77	
AT3G20340	12.43	6.87	12.62	6.35	**5.38**	9.11	**12.50**	9.28	
AT1G45145	88.06	76.22	99.21	76.27	**183.98**	200.61	**284.78**	239.67	THIOREDOXIN H-TYPE 5 (TRX5)
AT2G40880	132.22	404.08	132.16	425.35	**72.43**	810.08	**130.20**	915.61	CYSTATIN A (CYSA)
AT4G35770	61.08	1.97	74.01	0.92	**60.71**	1.25	**94.53**	1.75	SENESCENCE 1 (SEN1)
**Response to cold 13**									
AT3G05880	863.96	2330.43	998.16	2161.09	**1073.77**	7376.46	**1825.18**	7089.70	RARE-COLD-INDUCIBLE 2A (RCI2A)
AT3G05890	223.74	354.57	251.30	263.43	**315.29**	1035.68	**473.74**	1007.84	RARE-COLD-INDUCIBLE 2B (RCI2B)
AT2G01918	16.09	3.97	19.08	6.92	**7.75**	6.37	**14.13**	9.52	PSBQ-LIKE 3 (PQL3)
AT5G15970	1293.72	9451.25	1665.22	7411.15	**1831.46**	21357.40	**3277.76**	20403.00	KIN2
AT1G22190	43.89	18.66	58.27	14.53	**7.83**	6.34	**15.18**	6.59	
AT4G04330	142.02	165.32	141.53	164.43	**113.99**	259.42	**185.24**	253.06	HOMOLOGUE OF CYANOBACTERIAL RBCX 1 (RbcX1)
AT5G57560	305.30	54.83	359.91	73.13	**20.45**	11.05	**63.73**	14.18	TOUCH 4 (TCH4)
AT1G43160	9.03	19.30	10.60	26.91	**9.07**	24.42	**14.69**	28.17	RELATED TO AP2 6 (RAP2.6)
AT1G74520	58.57	84.66	58.01	87.35	**11.96**	42.01	**18.81**	46.92	HVA22 HOMOLOGUE A (HVA22A)
AT2G42530	417.64	2783.82	597.18	2630.63	**346.44**	4676.75	**793.67**	4564.97	COLD REGULATED 15B (COR15B)
AT2G40880	132.22	404.08	132.16	425.35	**72.43**	810.08	**130.20**	915.61	CYSTATIN A (CYSA)
AT5G37770	157.88	65.24	231.21	49.51	**90.70**	66.06	**165.41**	63.35	TOUCH 2 (TCH2)
AT5G42900	52.01	128.47	54.79	128.46	**12.94**	93.52	**21.11**	90.34	COLD REGULATED GENE 27 (COR27)
**Response to salicylic acid 11**									
AT3G50480	32.24	18.65	34.30	8.28	**21.03**	17.04	**44.58**	15.44	HOMOLOG OF RPW8 4 (HR4)
AT1G71030	90.61	35.23	86.31	54.96	**83.39**	58.17	**140.24**	66.76	MYB-LIKE 2 (MYBL2)
AT1G43160	9.03	19.30	10.60	26.91	**9.07**	24.42	**14.69**	28.17	RELATED TO AP2 6 (RAP2.6)
AT5G24530	13.01	20.18	15.53	17.11	**4.85**	12.46	**7.98**	11.04	DOWNY MILDEW RESISTANT 6 (DMR6)
AT3G28910	20.41	18.46	19.73	17.58	**7.29**	13.85	**12.26**	15.35	MYB DOMAIN PROTEIN 30 (MYB30)
AT2G05520	999.45	1110.65	1245.64	1156.65	**434.58**	657.44	**655.13**	764.78	GLYCINE-RICH PROTEIN 3 (GRP-3)
AT5G62470	14.41	8.22	19.56	8.21	**4.64**	4.77	**8.29**	4.81	MYB DOMAIN PROTEIN 96 (MYB96)
AT5G67300	55.84	66.86	81.58	60.21	**24.45**	43.21	**47.79**	42.08	MYB DOMAIN PROTEIN R1 (MYBR1)
AT1G73260	39.17	19.47	42.50	16.18	**13.20**	7.30	**25.18**	14.08	KUNITZ TRYPSIN INHIBITOR 1 (KTI1)
AT1G22640	30.98	9.96	39.36	11.10	**9.67**	4.41	**15.55**	5.94	MYB DOMAIN PROTEIN 3 (MYB3)
AT5G37260	20.24	149.19	24.55	145.71	**6.56**	70.44	**11.46**	72.01	REVEILLE 2 (RVE2)

**TABLE 7:** Partial classification data of up-transcribed genes in *atgcn1-1*, compared with that in WT under normal temperature conditions.

Local ID	gl1 CK	gl1 cold	gcn1-1 CK	gcn1-1 cold	gl1 CK _poly	gl1 cold _poly	gcn1-1 CK _poly	gcn1-1 cold _poly	Full_name
**Response to water deprivation 29**									
AT3G05640	**19.85**	4.31	**31.55**	6.44	6.07	2.90	8.01	2.93	
AT2G39800	**62.91**	80.34	**111.30**	88.60	21.65	21.94	23.47	24.43	DELTA1-PYRROLINE-5-CARBOXYLATE SYNTHASE 1 (P5CS1)
AT5G45340	**6.41**	5.06	**32.05**	9.61	1.13	0.60	3.03	0.99	CYTOCHROME P450, FAMILY 707, SUBFAMILY A, POLYPEPTIDE 3 (CYP707A3)
AT5G66400	**34.71**	19.59	**87.22**	16.23	70.70	12.06	88.62	10.96	RESPONSIVE TO ABA 18 (RAB18)
AT4G25480	**8.95**	14.57	**17.58**	14.96	4.72	18.27	15.50	16.88	DEHYDRATION RESPONSE ELEMENT B1A (DREB1A)
AT2G38470	**36.92**	35.26	**95.42**	33.70	17.57	11.55	31.75	11.13	WRKY DNA-BINDING PROTEIN 33 (WRKY33)
AT1G52400	**49.61**	38.88	**84.84**	37.31	4.01	1.79	5.27	3.03	BETA GLUCOSIDASE 18 (BGLU18)
AT5G59320	**24.68**	18.69	**47.42**	20.05	44.31	33.67	35.06	45.54	LIPID TRANSFER PROTEIN 3 (LTP3)
AT2G18050	**5.82**	1.70	**9.51**	2.74	4.87	0.90	6.61	1.87	HISTONE H1-3 (HIS1-3)
AT3G46620	**46.78**	21.42	**72.84**	15.87	14.65	10.27	37.56	9.81	RING AND DOMAIN OF UNKNOWN FUNCTION 1117 1 (RDUF1)
AT3G15500	**7.48**	1.58	**11.34**	2.10	2.22	0.47	3.78	0.76	NAC DOMAIN CONTAINING PROTEIN 3 (NAC3)
AT4G27410	**15.85**	29.94	**24.33**	32.47	5.60	10.35	6.52	10.06	RESPONSIVE TO DESICCATION 26 (RD26)
AT5G52300	**3.18**	24.88	**13.66**	34.55	4.96	6.70	9.23	7.32	LOW-TEMPERATURE-INDUCED 65 (LTI65)
AT2G35930	**9.58**	7.19	**14.72**	7.27	7.49	3.93	6.07	3.65	PLANT U-BOX 23 (PUB23)
AT3G02480	**4.72**	10.16	**31.79**	17.47	31.45	18.71	46.77	29.65	
AT3G19580	**7.33**	6.97	**19.17**	9.96	7.12	7.86	15.46	9.85	ZINC-FINGER PROTEIN 2 (ZF2)
AT5G15960	**32.28**	1518.85	**71.28**	1306.44	38.40	3731.74	153.14	4687.02	KIN1
AT4G25490	**5.26**	1.19	**16.29**	1.55	3.60	1.40	10.44	1.64	C-REPEAT/DRE BINDING FACTOR 1 (CBF1)
AT1G56600	**6.92**	7.01	**11.57**	4.40	2.65	3.21	2.69	2.70	GALACTINOL SYNTHASE 2 (GolS2)
AT4G39950	**29.44**	10.29	**47.37**	5.10	3.53	0.61	3.61	0.53	CYTOCHROME P450, FAMILY 79, SUBFAMILY B, POLYPEPTIDE 2 (CYP79B2)
AT1G27730	**45.75**	31.80	**114.02**	30.55	16.43	31.61	85.88	39.00	SALT TOLERANCE ZINC FINGER (STZ)
AT5G59310	**12.12**	25.52	**50.67**	52.75	24.04	27.34	26.11	62.39	LIPID TRANSFER PROTEIN 4 (LTP4)
AT3G14440	**5.42**	8.07	**16.02**	6.18	2.05	2.71	4.51	2.68	NINE-CIS-EPOXYCAROTENOID DIOXYGENASE 3 (NCED3)
AT5G05410	**8.88**	10.94	**14.89**	10.91	5.99	3.00	10.44	3.35	DRE-BINDING PROTEIN 2A (DREB2A)
AT3G45140	**122.44**	72.32	**206.37**	98.89	53.54	27.08	107.95	33.35	LIPOXYGENASE 2 (LOX2)
AT2G41010	**12.28**	13.75	**19.90**	16.46	6.63	15.56	12.42	17.06	CALMODULIN (CAM)-BINDING PROTEIN OF 25 KDA (CAMBP25)
AT2G46680	**14.09**	31.44	**24.70**	21.24	7.99	20.23	11.72	17.57	HOMEOBOX 7 (HB-7)
AT5G59550	**23.51**	14.24	**37.45**	14.43	9.31	6.75	10.17	7.39	RING AND DOMAIN OF UNKNOWN FUNCTION 1117 2 (RDUF2)
AT2G42540	**445.74**	9724.48	**768.31**	9132.78	254.30	15674.00	703.09	14885.20	COLD-REGULATED 15A (COR15A)
**Response to cold 19**									
AT4G02330	**8.10**	20.19	**14.21**	16.91	0.70	1.33	2.05	1.71	(ATPMEPCRB)
AT3G25770	**202.31**	173.83	**342.62**	233.35	120.73	180.21	247.39	214.19	ALLENE OXIDE CYCLASE 2 (AOC2)
AT5G52300	**3.18**	24.88	**13.66**	34.55	4.96	6.70	9.23	7.32	LOW-TEMPERATURE-INDUCED 65 (LTI65)
AT4G25490	**5.26**	1.19	**16.29**	1.55	3.60	1.40	10.44	1.64	C-REPEAT/DRE BINDING FACTOR 1 (CBF1)
AT2G33380	**6.76**	7.59	**15.44**	17.70	1.46	3.27	5.04	7.69	RESPONSIVE TO DESSICATION 20 (RD20)
AT5G15960	**32.28**	1518.85	**71.28**	1306.44	38.40	3731.74	153.14	4687.02	(KIN1)
AT4G25480	**8.95**	14.57	**17.58**	14.96	4.72	18.27	15.50	16.88	DEHYDRATION RESPONSE ELEMENT B1A (DREB1A)
AT2G38470	**36.92**	35.26	**95.42**	33.70	17.57	11.55	31.75	11.13	WRKY DNA-BINDING PROTEIN 33 (WRKY33)
AT4G25470	**17.27**	39.97	**37.57**	30.30	2.95	27.41	12.58	24.78	C-REPEAT/DRE BINDING FACTOR 2 (CBF2)
AT1G56600	**6.92**	7.01	**11.57**	4.40	2.65	3.21	2.69	2.70	GALACTINOL SYNTHASE 2 (GolS2)
AT5G47230	**20.48**	19.50	**59.84**	20.09	12.70	16.54	35.10	17.12	ETHYLENE RESPONSIVE ELEMENT BINDING FACTOR 5 (ERF5)
AT2G30250	**12.20**	22.18	**19.15**	26.00	6.35	8.35	11.37	8.87	WRKY DNA-BINDING PROTEIN 25 (WRKY25)
AT1G27730	**45.75**	31.80	**114.02**	30.55	16.43	31.61	85.88	39.00	SALT TOLERANCE ZINC FINGER (STZ)
AT3G14210	**199.00**	213.00	**364.24**	526.97	20.63	15.27	33.39	33.85	EPITHIOSPECIFIER MODIFIER 1 (ESM1)
AT3G45640	**90.77**	76.20	**135.99**	79.25	41.89	51.40	66.45	53.98	MITOGEN-ACTIVATED PROTEIN KINASE 3 (MPK3)
AT5G59820	**11.83**	29.44	**36.81**	37.05	6.52	29.78	22.15	30.10	RESPONSIVE TO HIGH LIGHT 41 (RHL41)
AT2G40140	**31.55**	52.88	**69.35**	47.59	13.36	22.90	20.99	21.69	(CZF1)
AT2G42540	**445.74**	9724.48	**768.31**	9132.78	254.30	15674.00	703.09	14885.20	COLD-REGULATED 15A (COR15A)
AT3G61190	**10.72**	12.27	**22.92**	11.24	2.54	8.35	11.76	9.77	BON ASSOCIATION PROTEIN 1 (BAP1)
**Response to salicylic acid 12**									
AT3G56400	**26.93**	9.18	**45.82**	3.60	9.90	4.94	30.69	5.57	WRKY DNA-BINDING PROTEIN 70 (WRKY70)
AT1G76930	**71.36**	166.58	**111.63**	152.17	25.07	28.10	31.38	51.39	EXTENSIN 4 (EXT4)
AT3G50060	**41.83**	15.73	**99.63**	13.69	10.33	9.48	27.62	8.89	MYB DOMAIN PROTEIN 77 (MYB77)
AT5G06320	**173.78**	94.50	**291.63**	65.24	27.13	16.87	50.71	19.73	NDR1/HIN1-LIKE 3 (NHL3)
AT1G21910	**23.40**	24.22	**34.77**	25.41	5.09	15.68	18.55	23.72	DEHYDRATION RESPONSE ELEMENT-BINDING PROTEIN 26 (DREB26)
AT4G23170	**9.98**	10.12	**15.53**	5.27	2.12	3.21	2.48	3.09	(EP1)
AT1G61340	**7.37**	11.50	**20.57**	7.26	2.26	7.13	5.64	4.69	F-BOX STRESS INDUCED 1 (FBS1)
AT1G18570	**14.91**	3.36	**25.20**	2.95	6.29	1.32	8.35	1.49	MYB DOMAIN PROTEIN 51 (MYB51)
AT2G40000	**168.95**	9.74	**307.14**	8.99	54.52	4.09	124.99	3.00	ORTHOLOG OF SUGAR BEET HS1 PRO-1 2 (HSPRO2)
AT2G33380	**6.76**	7.59	**15.44**	17.70	1.46	3.27	5.04	7.69	RESPONSIVE TO DESSICATION 20 (RD20)
AT1G80840	**10.27**	4.33	**60.81**	3.70	5.99	2.99	24.76	2.03	WRKY DNA-BINDING PROTEIN 40 (WRKY40)
AT3G61190	**10.72**	12.27	**22.92**	11.24	2.54	8.35	11.76	9.77	BON ASSOCIATION PROTEIN 1 (BAP1)

Due to the limited number of down-regulated in *atgcn1-1*, the classification may not make sense. Down-translated genes in *atgcn1-1* were found to be involved not only in plant development including flower development (11), post-embryonic development (5) and root hair elongation (5) (Table 8), but also in intracellular protein transport (14), protein folding (12), and endocytosis (8) (Supplementary Table 3).

**TABLE 8:** Partial Classification data of down-translated genes in *atgcn1-1*, compared with that in WT under temperature conditions.

Local ID	gl1 CK	gl1 cold	gcn1-1 CK	gcn1-1 cold	gl1 CK_poly	gl1 cold_poly	gcn1-1 CK_poly	gcn1-1 cold_poly	Full_name
**Flower development 11**									
AT5G60410	28.27	30.81	24.43	30.08	**15.25**	8.78	**10.02**	7.62	SIZ1
AT3G54610	6.86	6.92	7.00	6.75	**6.46**	2.26	**3.90**	2.48	HISTONE ACETYLTRANSFERASE OF THE GNAT FAMILY 1 (HAG1)
AT5G64960	30.15	25.21	28.10	31.74	**14.38**	5.44	**8.26**	4.73	CYCLIN DEPENDENT KINASE GROUP C2 (CDKC2)
AT2G28290	15.09	11.18	11.51	10.25	**13.12**	1.42	**8.12**	1.27	SPLAYED (SYD)
AT2G48160	18.62	22.58	15.67	24.64	**11.39**	5.53	**6.66**	5.11	
AT3G11540	12.96	17.17	12.64	20.09	**7.36**	5.42	**3.92**	5.75	SPINDLY (SPY)
AT4G32551	19.67	34.62	17.16	33.92	**10.58**	9.56	**6.53**	9.31	LEUNIG (LUG)
AT2G15530	11.32	7.13	10.07	7.78	**8.30**	2.37	**4.64**	2.25	
AT1G30330	18.41	38.03	14.50	36.57	**9.55**	10.79	**6.09**	9.68	AUXIN RESPONSE FACTOR 6 (ARF6)
AT1G79000	13.79	14.91	11.61	14.93	**8.61**	2.18	**5.07**	1.91	HISTONE ACETYLTRANSFERASE OF THE CBP FAMILY 1 (HAC1)
**Post-embryonic development 5**									
AT5G20490	22.26	22.51	17.87	21.14	**11.36**	2.94	**6.69**	2.38	XIK
AT1G50030	11.84	12.34	9.32	12.79	**7.25**	1.35	**4.63**	1.24	TARGET OF RAPAMYCIN (TOR)
AT3G51550	95.31	117.14	84.45	114.25	**12.80**	4.49	**7.08**	3.83	FERONIA (FER)
AT3G42170	21.59	21.77	17.49	18.77	**10.07**	8.03	**6.59**	7.26	
AT3G13300	24.01	42.51	20.68	38.13	**13.69**	9.90	**8.73**	9.15	VARICOSE (VCS)
**Root hair elongation 5**									
AT5G20490	22.26	22.51	17.87	21.14	**11.36**	2.94	**6.69**	2.38	XIK
AT5G13010	21.59	14.68	20.28	15.96	**11.90**	3.10	**7.44**	2.71	EMBRYO DEFECTIVE 3011 (EMB3011)
AT4G30160	22.66	25.65	21.63	22.64	**13.21**	4.56	**8.49**	4.11	VILLIN 4 (VLN4)
AT5G09810	315.39	290.08	252.95	253.12	**151.60**	148.71	**100.92**	142.14	ACTIN 7 (ACT7)
AT5G43900	18.25	18.59	15.74	20.36	**8.80**	2.74	**5.76**	3.23	MYOSIN 2 (MYA2)

### Kozak Sequence and uORFs Affect Translation Selection Under Normal Temperature Conditions

As shown in Figure 3A, WT plants have a low level of eIF2α phosphorylation in normal temperature conditions. The low level of eIF2α phosphorylation were showed to regulate expression of specific genes (Tables 6-8 and Supplementary Table 3). We wonder how the low level of eIF2α phosphorylation regulate protein translation, so we further analyzed the initiation codon and uORFs of the up-translated and down-translated genes in *atgcn1-1* under normal temperature conditions.

The percentage of up-translated and down-translated transcripts with strong initiation codon in *atgcn1-1* is respectively 45.6% and 41.8%, both are higher than 34% of total genes (Table 9). Meanwhile, the percentage of up-translated and down-translated transcripts with adequate initiation codon in *atgcn1-1* are respectively 45.6% and 41.8%, both are higher than 34% of total genes. On the other hand, the percentage of up-translated and down-translated transcripts with weak initiation codon or without 5′-UTR are lower than that of total genes. The results show that the transcripts with strong or adequate initiation codon have the preference to be regulated by GCN1 mediated eIF2α phosphorylation, which is consistent with the preference of cold-AtGCN1 mediated eIF2α phosphorylation.

**TABLE 9:** The frequency of initiation codon and uORFs for up-translated and down-translated genes in *atgcn1-1*, compared with WT, under normal temperature conditions.

	Transcripts with strong initiation codon (%)	Transcripts with adequate initiation codon (%)	Transcripts with weak initiation codon (%)	Transcripts without 5′-UTR	uORF frequency in mRNA (%)
Up	45.6	48.9	4.9	5.9	63.1
Down	41.8	49.2	7.1	2.0	70.3
Total genes	34.0	44.5	10.6	10.8	51.2

We next analyzed the uORFs of up-translated and down-translated genes in *atgcn1-1*, compared with WT. The uORF frequency of up-translated and down-translated transcripts is individually 63.1% and 70.3%, both are higher than that in total genes (51.2%). The results demonstrate that the transcripts with uORFs have the preference to be regulated by GCN1 mediated eIF2α phosphorylation in normal temperature conditions, which also coincides with the preference of cold-AtGCN1 up and down-translated genes by cold-induced eIF2α phosphorylation.

## Discussion

In *Arabidopsis*, GCN2 is the only known eIF2α kinase to phosphorylate eIF2α. Additionally, eIF2α can’t be phosphorylated under amino acid starvation in *gcn2*-knockout mutants (Lageix et al., 2008). In our previous study, we have cloned AtGCN1 to demonstrate that AtGCN1 interacts with GCN2 to phosphorylate eIF2α upon cold stress (Wang et al., 2017). These evidence collectively indicate that the phosphorylation of eIF2α is defective in *atgcn1* mutants and AtGCN1-GCN2 mediated eIF2α phosphorylation is essential for cold acclimation.

In this study, we further investigated the function of AtGCN1 in *Arabidopsis*. Firstly, we found that *atgcn1* mutants flowered later than the WT (Figure 1). Secondly, we discovered that plant growth and seed development were arrested in *atgcn1* mutants (Figure 2). FLC, a negative regulator of flower development, could be attributed to this observation as it was up-regulated in *atgcn1-1* compared to the WT (Figure 2B and Supplementary Table 1).

Since the phosphorylation of eIF2α is defective in *atgcn1*, the translation of protein was proposed to be in an enhanced level in *atgcn1*. The defects noted in plant growth and seed development of mutant *atgcn1*, is opposite with the improved growth of AtTOR-overexpressing plants (Deprost et al., 2007), although the loss of eIF2α phosphorylation in *atgcn1* as well as AtTOR-overexpression may promote protein translation (Wang et al., 2017). The results show that enhanced protein translation doesn’t associate with vigorous growth and AtGCN1 play roles in the growth at both vegetative and reproductive stages.

Thus far, AtGCN1 has been observed to interact with only eIF2 kinase GCN2 to phosphorylate eIF2α upon cold stress (Wang et al., 2017). However, it is still not clear how AtGCN1 mediated eIF2α phosphorylation upon cold stress affects transcription and protein translation in plants. Therefore, we performed sequencing on the total and polysomal RNA of *atgcn1* mutants after cold treatments to further examine the function of eIF2α phosphorylation mediated by AtGCN1.

Hierarchical clustering analysis of cold-AtGCN1 regulated genes at both transcriptional and translational levels demonstrates that there are alternative pathways, independent of eIF2α phosphorylation, involved in regulating gene expression at both transcriptional and translational levels (Figures 3, 4). In mammals, concerted activation of AMPK (AMP-activated protein kinase), inhibition of TORC1 (TARGET OF RAPAMYCIN complex 1), phosphorylation of eIF2α and other signals may inhibit protein synthesis in parallel to ensure cellular survival under low temperature and endoplasmic reticulum (ER) stress (Hofmann et al., 2012; Guan et al., 2014). In *Arabidopsis*, there are at least two pathways required to suppress protein translation under cold stress, including the inhibition of TOR1 and eIF2α phosphorylation (Wang et al., 2017). The RNA sequencing results confirm that AtGCN1 mediated eIF2α phosphorylation is not the only way to monitor gene transcription and protein translation in cold stress.

Importantly, the RNA sequencing results demonstrated that AtGCN1-mediated eIF2α phosphorylation upon cold stress selectively regulates gene expression at both transcriptional and translation levels. The classifications of cold-AtGCN1 regulated genes show that AtGCN1-mediated eIF2α phosphorylation upon cold stress selectively regulate the transcription or translation of genes in response to blue light, hormone stimulus, toxin, wounding, salt stress, osmotic stress and organic substance; suggesting that AtGCN1 has a functional role in abiotic or biotic stress, in addition to cold stress. Moreover, AtGCN1-mediated eIF2α phosphorylation upon cold stress selectively regulates the gene expression involved in amino acid biosynthesis, photosynthesis, cell wall organization, protein transport and localization, lipid biosynthesis, gene transcription, macroautophagy, proteolysis and cell death.

In normal temperature conditions, the low level of eIF2α phosphorylation in WT is always detected (Figure 3A), so we infer that the low level of eIF2α phosphorylation may regulate gene expression. We compared the expression difference between *atgcn1-1* and WT in normal temperature conditions and a lot of genes were filtered out (Supplementary Table 3). The results indicate that the low level of eIF2α phosphorylation in normal temperature conditions can selectively regulate gene expression at both transcriptional and translational level, as well as the cold-induced high level of eIF2α phosphorylation in cold stress conditions.

Interestingly, we found that cold-AtGCN1 induced or inhibited genes in WT at both transcriptional and translational levels were mostly induced or inhibited in *atgcn1-1* in normal temperature conditions, compared with that in WT (Tables 1–4 and Supplementary Tables 1, 2). The results indicate that the high level of eIF2α phosphorylation in cold stress conditions and the low level of eIF2α phosphorylation in normal temperature conditions play opposite roles in the regulation of gene expression. In other words, the high level of eIF2α phosphorylation promotes or inhibits the expression of specific genes at both transcriptional and translational levels, while the low level of eIF2α phosphorylation oppositely regulates the expression of these genes.

The classifications of up-regulated genes in *atgcn1-1* in normal temperature conditions show that AtGCN1 negatively regulates the transcription or translation of genes involved in biotic stress, abiotic stress and hormone signaling (Tables 6, 7). The results indicate that AtGCN1 mediated eIF2α phosphorylation should respond to a lot of stimulus, in addition to cold stress, which is consistent with the conclusion of cold-AtGCN1 up-regulated genes (Tables 2, 4). Moreover, a lot of genes in plant development were found to be down-translated in *atgcn1-1* in normal temperature conditions (Table 8 and Supplementary Table 3), including flower development, post-embryonic development, cell wall organization, primary shoot apical meristem specification, root hair elongation, cell growth, cell division and seed germination.

The down-translated genes of *atgcn1-1* in plant development suggest that eIF2alpha phosphorylation play an important role in plant growth. Firstly, we found that *atgcn1* mutants flowered later than the WT (Figure 1). Meantime, we discovered that plant growth and seed development were arrested in *atgcn1* mutants (Figure 2). The down-translated genes of *atgcn1-1* in plant development may explain the late flowering, arrested growth and seed defect noticed in *atgcn1* mutants.

We analyzed the initiation codon and uORFs of cold-AtGCN1 down-translated, cold-AtGCN1 up-translated genes and down/up-translated genes in *atgcn1-1* in normal temperature conditions (Tables 5, 9). These results demonstrate that eIF2α phosphorylation results in the translation preference to transcripts with strong or adequate initiation codon. In other words, transcripts with a weak initiation codon or without 5′-UTR has less opportunity to be regulated by eIF2α phosphorylation than ones with a stronger initiation codon, which is the first report to our knowledge. Moreover, the uORF analysis of these genes shows that AtGCN1 mediated eIF2α phosphorylation may regulate the translation preference to transcripts with uORFs, which is different from the function of eIF2α phosphorylation in yeast and mammalians.

In yeast and mammalians, eIF2α phosphorylation specifically increases the translation of GCN4 or ATF4 with uORFs (Mueller and Hinnebusch, 1986; Vattem and Wek, 2004). Due to pathogen-triggered eIF2α phosphorylation, *Arabidopsis* TBF1 is supposed to be specifically translated through its two uORFs (Pajerowska-Mukhtar et al., 2012; Lokdarshi et al., 2020). However, TBF1 is not regulated in the data. The most likely cause is that TBF1 is specifically induced by pathogen infection, not by cold stress treatment used here.

In addition, among the down-translated genes in *atgcn1-1* under normal temperature conditions, we found that lots of chloroplastic and mitochondrial genes were down-translated in *atgcn1-1*, compared with that in WT (Supplementary Table 3). The results suggest that the lack of eIF2α phosphorylation in *atgcn1* may repress the translation of organellar genes, which need be explored in the future.

In this work, a further investigation of AtGCN1 was achieved. Total and polysomal RNA sequencing of *atgcn1-1* shows that eIF2 phosphorylation mediated by AtGCN1 selectively regulates gene expression at both transcriptional and translational levels and Kozak sequence and uORFs of transcripts affect translation selection. Moreover, mutations of *atgcn1* impairs flowering time, plant growth and seed development, which reflecting the affection of the expression alteration in *atgcn1-1*. All results show that AtGCN1 mediated eIF2 phosphorylation selectively regulates gene transcription and protein translation in *Arabidopsis.*

## Data Availability Statement

The datasets presented in this study can be found in online repositories. The name of the repository and accession number can be found below: Genome Sequence Archive in National Genomics Data Center, China National Center for Bioinformation/Beijing Institute of Genomics, Chinese Academy of Sciences, https://bigd.big.ac.cn/gsa/, CRA003757.

## Author Contributions

HZ designed the research. XC, KG, LW, ML, ZL, DZ, LD, and XL performed the experiments. HZ, WW, and WY analyzed the sequencing data. HZ and J-KZ wrote the manuscript. All authors contributed to the article and approved the submitted version.

## Conflict of Interest

The authors declare that the research was conducted in the absence of any commercial or financial relationships that could be construed as a potential conflict of interest.

## Footnotes

1 http://david.abcc.ncifcrf.gov/

## Supplementary Material

Supplementary Table 1 Cold-AtGCN1 regulated genes at transcriptional levels.

Supplementary Table 2 Cold-AtGCN1 regulated genes at translational levels.

Supplementary Table 3 Regulated genes in *atgcn1-1* in normal temperature conditions.

Supplementary Table 4 Primers used for qRT-PCR.
